# Overwintered *Drosophila suzukii* Are the Main Source for Infestations of the First Fruit Crops of the Season

**DOI:** 10.3390/insects9040145

**Published:** 2018-10-22

**Authors:** Aurore D. C. Panel, Laura Zeeman, Bart J. van der Sluis, Peter van Elk, Bart A. Pannebakker, Bregje Wertheim, Herman H. M. Helsen

**Affiliations:** 1 Groningen Institute for Evolutionary Life Sciences, University of Groningen, Nijenborgh 7, 9700 CC Groningen, The Netherlands; a.d.c.panel@rug.nl (A.D.C.P.); b.wertheim@rug.nl (B.W.); 2 Wageningen University & Research, Laboratory of Genetics, PO Box 16, 6700 AA Wageningen, The Netherlands; bart.pannebakker@wur.nl; 3 Wageningen University & Research, Laboratory of Entomology, PO Box 16, 6700 AA Wageningen, The Netherlands; laura.zeeman@wur.nl; 4 Wageningen University & Research, Field Crops, 6670 AE Zetten, The Netherlands; bart.vandersluis@wur.nl (B.J.v.d.S.); peter.vanelk@wur.nl (P.v.E.)

**Keywords:** *Drosophila suzukii*, alternative host, seasonal biology, phenotypic plasticity, integrated pest management

## Abstract

The mechanisms allowing the widespread invasive pest *Drosophila suzukii* to survive from early spring until the availability of the first fruit crops are still unclear. Seasonal biology and population dynamics of *D. suzukii* were investigated in order to better understand the contribution of the early spring hosts to the infestation of the first fruit crops of the season. We identified hosts available to *D. suzukii* in early spring and assessed their suitability for the pest oviposition and reproductive success under field and laboratory conditions. The natural infestation rate of one of these hosts, *Aucuba japonica*, was assessed over springtime and the morphology of the flies that emerged from infested *A. japonica* fruits was characterized under field conditions. Then, these findings were correlated with long-term monitoring data on seasonal reproductive biology and morphology of the pest, using a cumulative degree-days (DD) analysis. Field sampling revealed that overwintered *D. suzukii* females were physiologically able to lay eggs at 87 DD which coincided with the detection of the first infested early spring hosts. The latter were continuously and increasingly infested by *D. suzukii* eggs in nature from early spring until the end of May, in particular *Aucuba japonica*. Individuals emerged from most of these hosts were characterized by a poor fitness and a rather low success of emergence. In the field, only few summer morphs emerged from naturally infested *A. japonica* fruits around the end of May-beginning of June. However, field monitoring in orchards revealed that *D. suzukii* individuals consisted solely of winter morphs until mid-June. These observations indicate that overwintered *D. suzukii* females are the predominant source for the infestations in the first available fruit crops of the season. We discuss these findings in the context of possible pest control strategies.

## 1. Introduction

In the late 2000s, the Asian *Drosophila suzukii* (Matsumura; Diptera: Drosophilidae), invaded Europe and North America, infesting a wide range of ripening fruits and causing significant economic losses [1,2,3,4,5,6,7]. In the Netherlands, *D. suzukii* was first recorded in 2012 [8]. Since then, the number of *D. suzukii* fruit flies has increased and caused substantial damage to the Dutch soft fruit industry [9]. Most existing pest management tools are inadequate against *D. suzukii* and current control efforts rely mainly on the use of broad-acting pesticides [6,10,11,12].

The best prospects for controlling infestations of this invasive pest may come from integrated pest management (IPM) including cultural and technical methods, chemical, biotechnical, and biocontrol approaches [1,6]. IPM strategies are most likely to succeed when they can build on fundamental knowledge of the pest species. Specifically, knowledge of the pest seasonal biology could help to identify periods of vulnerability in *D. suzukii*’s life cycle. Such information is of crucial importance for all stakeholders of the soft-fruit sector [13,14,15], and also to make informed decisions on control measures to reduce further population buildup [13,16,17,18].

In temperate climates, several long-term monitoring studies have shown a sharp decline in the number of *D. suzukii* captures from winter onwards [19,20]. In Europe, this population decline lasts until late spring, when the pest is recorded in massive numbers in cherry crops, which are generally the first commercial fruits available to *D. suzukii* [13,19]. This field monitoring pattern can be explained by the fact that *D. suzukii* populations undergo two bottleneck periods [13]. The first bottleneck period relates to winter time when the pest encounters harsh environmental conditions. *Drosophila suzukii* can respond to this seasonal change through a range of physiological and morphological adaptations that enhance its survival to some extent [21]. Adults of *D. suzukii* enter a state of reproductive dormancy during winter months; most females captured in the field during this period have undeveloped ovaries [21,22,23,24]. Males are generally scarce at this time of the year and produce very few sperm [13]. In addition, *D. suzukii* is able to develop a specific morphology. As summer progresses towards winter, flies develop darker pigmentation and longer wings [1,21,25]. Such flies are characterized as winter morphs, as opposed to summer morphs that are present during summertime, and have a higher cold tolerance [21,26]. Seasonal morphologies are irreversible whereas reproductive diapause can be ended when climatic conditions become more favorable to the pest reproduction [24]. Thus, adult winter morph females that mated in autumn and overwintered, start bearing mature eggs in early spring.

The second bottleneck period occurs in early spring, when winter *D. suzukii* survivors form small populations and have restricted availability of host plants with fruits that can be used for food and reproduction [13]. These early spring host plants, often referred to as “non-crop hosts”, “non-cultivated hosts”, “alternative hosts ”, or “wild hosts”, to differentiate them from crops, include plants found in nature and ornamental species in parks and gardens [15,18,27,28]. Whilst *D. suzukii* is extremely polyphagous, can develop in many fruit species, and switch between these depending on the fruit seasonality [27], it remains unclear what the contribution is of the early spring hosts to the seasonal buildup of the population. As these non-crop host plants might constitute the starting point of the next generations of fruit flies, many regional surveys have been conducted to identify them. In Europe and North America, extensive field surveys have reported more than a hundred host species in which the pest can develop [15,18,27,28,29]. In Germany, mistletoe is thought to be one of the first reproductive hosts of *D. suzukii* in early spring [18]. In Italy, ivy berries are continuously infested by the pest from the beginning of April until the end of May. Although the fruit flies emerging from these berries have poor fitness, they hatch in the early season and could potentially attack the first available commercial crops [13].

In this study we investigated the seasonal biology of *D. suzukii* to identify the factors that regulate their population ecology in early spring. Our objective was to determine to what extent *D. suzukii* emerging from wild host plants in early spring contribute to the populations that infest the first fruit crops of the season, in our case, commercial cherry crops. We specifically (1) identified hosts available to *D. suzukii* in early spring and assessed their suitability for the pest oviposition and reproductive success under field and laboratory conditions; (2) evaluated the natural infestation rate of one of these hosts, *Aucuba japonica*, over springtime and scored the morphology of the flies that emerged from infested *A. japonica* fruits under field conditions; (3) then, these findings were correlated with long-term monitoring data on seasonal reproductive biology and morphology of the pest by using a temperature-related population model optimized for *D. suzukii* by [30].

## 2. Materials and Methods

The study took place in the central Netherlands (51.7460077–52.0333300 N, 4.7083300–5.6763900 E; elevation above sea level: 0.11–20 m). All laboratory experiments were performed in a climate chamber at 20.8 ± 0.6 °C, 16:8 light:dark photoperiod, 70% RH. Plastic containers (Ø52 mm, 125 mL) used for all trials were furnished with a 2 cm layer of humid oasis floral foam, covered with a fine mesh netting and sealed with a screwcap lid with a 4 cm² opening for ventilation.

### 2.1. Identification and Natural Infestation of Early Spring Hosts

Potential host plants were surveyed in the field in early spring for *D. suzukii* infestation. Six plant species were examined in 2016: *Aucuba japonica*, *Skimmia japonica*, *Cotoneaster* spp., *Elaeagnus x ebbingei*, *Hedera helix*, and *Viscum album* (Table S1). The selection was based on a field survey performed in the same region by [15] and on the results of other studies [18,27]. For all fruit species an estimation of the emergence rate of *D. suzukii* adults was performed. All field-collected fruits were ripe. Fruits were kept in containers in the laboratory for several weeks and emerging *D. suzukii* adults were collected and counted, following the methodology developed by [15].

Based on the results obtained in 2016, five host plant species were monitored in 2017, i.e., *A. japonica*, *E. x ebbingei*, *S. japonica*, *H. helix*, and *V. album*. The presence and phenology of these fruit species in the field was recorded every month to determine the temporal availability of host plants with respect to population dynamics of the pest (Table 1). In order to determine the infestation rate of the above mentioned fruit species by *D. suzukii* eggs, over 200 fruits of each species were randomly collected. This procedure was performed at two time points and at different sites (Table S2). After collection, the fruits were examined for *D. suzukii* egg presence with a stereomicroscope. The number of eggs laid on each fruit was determined by checking for egg filaments. In order to monitor fly emergence and estimate egg-to-adult survival, infested fruits were placed in plastic containers and stored in a climate chamber. The containers were regularly inspected and emerging *D. suzukii* adults collected and recorded.

### 2.2. Natural Infestation of A. japonica by D. suzukii over Time and Phenotype of Emerged Adults

A field trial was conducted to assess the natural infestation rate of *A. japonica* by *D. suzukii* eggs from early to late spring. The phenotype of the flies that emerged from infested *A. japonica* fruits under natural conditions during this experimental period was scored.

*Aucuba japonica* was used as early spring host, because this plant produces fruits from winter onwards and is very common in the study area. Furthermore, *D. suzukii* readily accepted this host for egg-laying in 2016: out of 16 locations investigated, 15 hosted infested plants. In a preliminary laboratory experiment, the egg-to-adult survival of *D. suzukii* in *A. japonica* fruits was 23% [31].

Fruits of *A. japonica* were collected weekly from 15 March until 30 May 2017. Fruits were sampled from three municipalities: Wageningen, Gouda, and Zederik, from one or several collection sites per municipality (Table S3). Each site was separated by a minimum distance of 100 m. Each week, the fruits were picked from three to eight sites and stored in paper bags. On average, between 40 and 70 fruits per site were randomly collected weekly, to have around 300 fruits in total.

**Figure 1:**
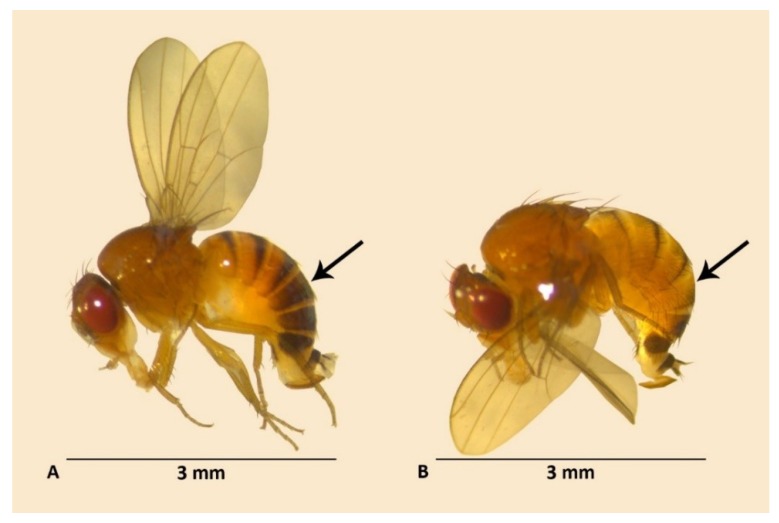
Characteristic *D. suzukii* females of the two phenotypes: (**A**) winter morph female and (**B**) summer morph female. Arrow points toward the fourth abdominal segment which is completely melanized in adult females displaying a winter phenotype. In males, this difference occurs on the third abdominal segment. Photographs: A.D.C.P.

After collection, the fruits were examined for *D. suzukii* egg presence with a stereomicroscope, checking for egg filaments. Approximately half of the infested fruits were incubated in plastic containers outside, under natural conditions, whereas the other half were stored in plastic containers in the climate chamber of the laboratory. The containers were checked regularly and emerging *D. suzukii* adults were recorded. The offspring obtained from the fruits that had been incubated outside were stored at 4 °C in ethanol 70% and assessed 3–4 weeks later under a stereomicroscope to determine their phenotype. A *D. suzukii* fruit fly can be classified into summer, winter or intermediate morph based on the degree of abdominal melanization. Summer morph females have only a thin dark stripe at the end of the fourth tergite whereas the 4th and 5th tergites of winter morph females are dark brown to black (Figure 1). In males, this difference occurs on the 3rd tergite which is melanized in winter morphs and yellow with a thin black stripe in summer morphs. Winter-morph males and females have also a darker thorax compared with summer morph individuals. Intermediate morphs have an in-between phenotype [32].

### 2.3. Performance of D. suzukii on Early Spring Hosts in Controlled Conditions

No-choice trials were performed in the laboratory in order to assess oviposition preference and egg-to-adult survival of *D. suzukii* on the identified early spring hosts. Adult *D. suzukii* were obtained from the laboratory-reared colony that had been started in 2013 from about 100 individuals, collected in France (GPS coordinates: 43.754059 N, 4.4595 E). All flies had a summer phenotype and were provided with an artificial diet (DTS070 Drosophila Quick Mix Medium, Blades Biological Ltd., Edenbridge, UK) that served as both a food source and an oviposition medium.

Fruits of *A. japonica*, *E. x ebbingei*, *S. japonica*, *H. helix*, and *V. album* were collected from different sites on 8 May 2017 (Table S2). *Vaccinium* spp. organic fruits (blueberries) originating from Spain (Huelva) were bought from a supermarket in the Netherlands as a control. All fruits were checked under a stereomicroscope in order to make sure they had not been damaged or naturally infested by *D. suzukii*. The experimental unit was a plastic container fitted with a 5% honey-water solution (Melvita organic honey). In order to minimize desiccation of the fruit species, the containers were maintained in relatively high humidity conditions (70% RH) and furnished with a layer of humid oasis floral foam throughout the assay. The number of fruits per container varied with fruit species in order to account for the differences in fruit size and to offer the flies the same amount of surface area available for oviposition. Species of *A. japonica* and *Vaccinium* spp. had six fruits each per container, *E. x ebbingei* species had 12 fruits per container and *S. japonica*, *H. helix* and *V. album* had 14 fruits per container. Seven replicates per fruit species were set up in the climate chamber of the laboratory. In each container, seven two-week-old *D. suzukii* females and three two-week-old *D. suzukii* males were released. After 48 h, the flies and the honey-water solution were removed and the number of eggs laid was counted by checking for egg filaments using a stereomicroscope. The fruits were then transferred back to their original container and maintained under rearing conditions (20.8 ± 0.6 °C, 16:8 light:dark photoperiod, 70% RH) until the flies emerged. The containers were checked for fly emergence for approximately 3 weeks. The developmental time of flies was also recorded. The size of *D. suzukii* individuals that emerged from the various fruit species was estimated by measuring their wing length, according to the methodology developed by [33], using a Dino-Lite digital microscope and the DinoCapture 2.0 software (Dino-Lite Europe, Naarden, The Netherlands).

### 2.4. Field Monitoring Program

Individuals of *D. suzukii* were captured in an area-wide monitoring program, from 2016 to 2017, using nine traps in three sites all located within a 10 km radius around Wageningen. Each of these three sites corresponds to a distinct habitat. The first habitat is characterized by commercial cherry orchards and private gardens located along a busy trunk road. The second habitat is made of a vineyard enclosed by woods and shrubs of varying species and the third one is situated in the applied research station of Randwijk, surrounded by berry and cherry orchards (Table S4). Traps were commercially available Droso-Traps (Biobest, Westerlo, Belgium) baited with around 200 mL of Dros’Attract (Biobest, Westerlo, Belgium). The trap catches were collected weekly or biweekly during 2016 and 2017. Caught fruit flies were stored at 4 °C in 70% ethanol until further processing.

#### 2.4.1. Ovary Dissection

**Figure 2:**
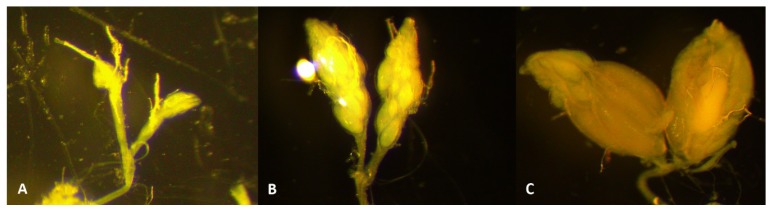
Ovarian development of *D. suzukii* female: (**A**) unripe ovarioles, (**B**) maturing eggs, (**C**) mature eggs (magnification: 40×). Photographs: A.D.C.P.

From the samples that were collected during the whole year of 2017, at least 35 captured *D. suzukii* females were dissected for each month in a phosphate-buffered saline solution during the whole year of 2017 in order to determine the period of reproductive diapause and the resumption of oogenesis. They were sampled from across all monitoring locations (Table S4) and categorized based on their ovarian development (Figure 2) [18,23,34] in four categories: indiscernible ovarioles; unripe ovarioles; maturing eggs; and mature eggs. Some females’ ovaries were classified as “damaged” due to dissection issues.

#### 2.4.2. Phenotype Assessment

For each month, from May 2016 until December 2017, the phenotype of at least 36 captured *D. suzukii* males and females (i.e., summer or winter morph) was scored in order to identify the switch point from winter morph to summer morph populations for two consecutive years. These individuals were equally sampled from across all monitoring locations. The fruit flies were classified into summer, winter, or intermediate morphs based on the degree of abdominal melanization, using a stereomicroscope.

### 2.5. Temperature Model

The “single sine method” of Degree-Days (DD) calculation [35] was used to estimate DD accumulation for *D. suzukii* during 2016 and 2017. Calculations of heat accumulation started on 1 January. This model was based on a temperature-related population model optimized for *D. suzukii* by [30]. We only used the lower threshold of 7.2 °C, because the upper threshold of 30 °C was not relevant for the spring climate in the Netherlands.

The thermal constant for developmental time from egg-to-adult was also taken from this study and was 208 DD [30]. This parameter was used to predict the expected switch point from winter morph to summer morph populations based on the first collection of infested early host fruits. It was also used to determine the oviposition date of the actual first generation of summer-morph flies captured in the field. The temperatures were obtained from the Wageningen University and Research weather station “Veenkampen” in Wageningen (51.981216 N, 5.620416° E).

### 2.6. Data Analysis

R (R development Core Team 2017) was used for statistical analyses. A *p*-value of < 0.05 was interpreted as statistically significant. The suitability of alternative host plants for *D. suzukii* oviposition in the field was examined with a generalized linear model (GLM). For each plant species, the collected fruits from all sites were aggregated per collection date and the percentage of infested fruits was represented as a fraction of the whole collected sample. In this model, the infestation rate of the fruits by *D. suzukii* eggs was the response variable.

In the no-choice laboratory trials, the effect of fruit species on the number of *D. suzukii* eggs laid and adults emerging from fruits were analyzed with GLMs. In the “egg-model”, the number of *D. suzukii* eggs was the response variable. In the “adult-model”, the response variable was the egg-to-adult survival of *D. suzukii*, calculated as the ratio between the total number of eggs laid on the incubated fruits and the number of flies emerged from these fruits. The combined effect of fruit species and number of eggs on the developmental time was also examined, using a linear model (LM). In this “development-model”, the median developmental time (in number of days) per replicate was the response variable. The assumptions of normality were validated and the significance of terms was tested with *F*-tests and the function drop1. Wing length data were analyzed with a two-way ANOVA. All models were simplified by removing non-significant interactions and post-hoc comparisons of means were performed with Tukey tests.

The weekly collected *A. japonica* fruits from all sites were aggregated per collection date and the percentage of infested fruits was represented as a fraction of the whole collected sample for each week. The reproductive status and phenotype of the field-collected *D. suzukii* individuals from all sites were respectively grouped per week and the percentage of each maturation category or phenotype was represented as a fraction of the whole analyzed population for that corresponding week. The reproductive biology is presented together with the minimum and maximum daily temperatures in the area.

## 3. Results

### 3.1. A. japonica Is Highly Infested by D. suzukii in Nature, but with a Low and Variable Egg-to-Adult Survival

Four non-crop plants were identified as early spring host plants of *D. suzukii* in spring 2016. Indeed, the pest could complete its lifecycle on *S. japonica*, *E. x ebbingei*, *A. japonica*, and *V. album*. In contrast, no *D. suzukii* adults emerged from *Cotoneaster* spp. and *H. helix* (Table 2). Although collected from March onwards, *V. album* started to yield *D. suzukii* adults only when fruits where collected after mid-May (Table S5). *Hedera helix* was not identified as a host for *D. suzukii* in 2016 but we decided to include it in the second part of the study because this plant species had been shown to be an early spring host in Italy [13] and was very common in the study area.

Both fruit species and collection date significantly affected the infestation rate of the fruits in 2017 (GLM, binomial distribution: *χ*² _(4)_ = 5239, *p* < 2 × 10^−16^ for fruit species and *χ*² _(1)_ = 28.2, *p* = 1 × 10^−7^ for collection date), but the interaction term between the fruit species and the collection date was not significant (GLM, binomial distribution: *χ*² _(3)_ = 4.7, *p* = 0.19). Each plant species was significantly different from the others with respect to the infestation rate. Fruits of *A. japonica* were the most heavily infested (Table 3). Fruits of *V. album* were not infested by the pest. From the fruits collected on 17 April, emergence of adult flies was only obtained in *A. japonica* species and the egg-to-adult survival was very low. On 8 May, *D. suzukii* adults emerged from *A. japonica* and *E. x ebbingei* with a higher egg-to-adult survival (Table 3).

### 3.2. D. suzukii Infest A. japonica from Early to Late Spring and a Few Summer-Morph Flies Emerge from This Host

**Figure 3:**
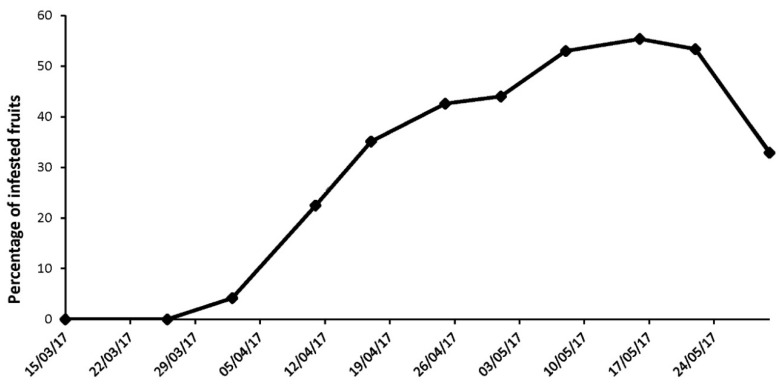
Infestation rate of collected *A. japonica* fruits by *D. suzukii* eggs under natural conditions from early to late spring 2017. Results are presented as the percentage of collected fruits naturally infested by *D. suzukii* eggs at each collection date. For each collection date, over 200 fruits were randomly sampled.

From the beginning of April (104–106 DD) until 30 May (393 DD), *A. japonica* fruits were infested by *D. suzukii* (Figure 3). The infestation rate increased over time and peaked at 257 DD (16 May) with more than 55% of infested berries. From the fruits that had been incubated outdoors under natural conditions between 10 April and 29 May, a few flies emerged between 27 May (358 DD) and 13 June (516 DD) (Table 4). Of these emerged flies, 97% were summer morph individuals. The others had an intermediate phenotype.

### 3.3. Laboratory Assays Confirm the Field Observations

**Figure 4:**
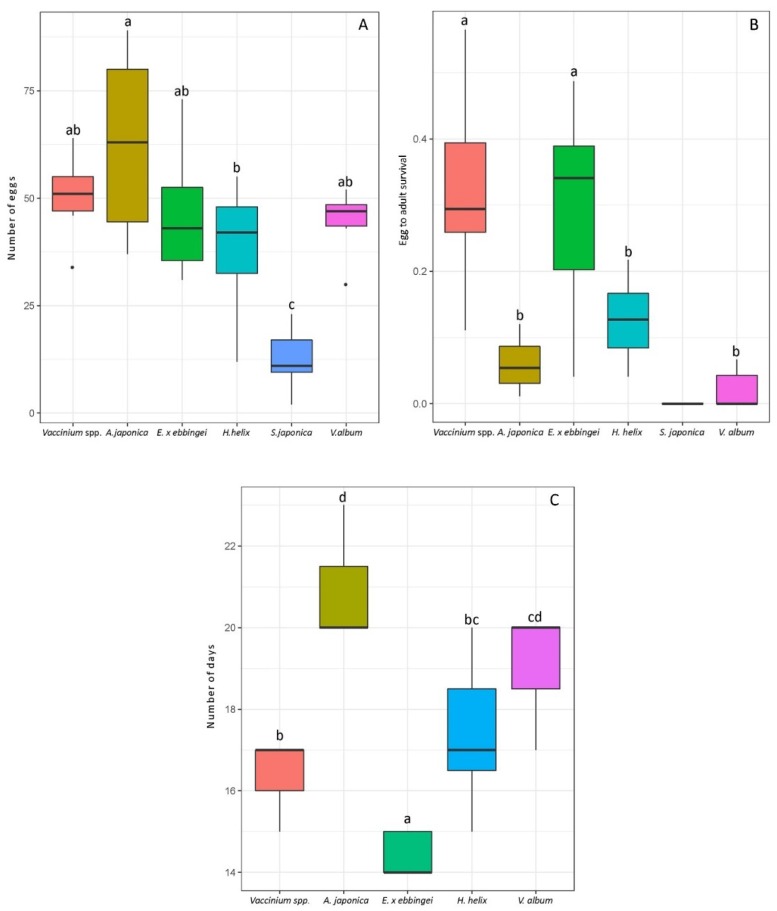
Performance of *D. suzukii* on various early spring hosts and on blueberries in no-choice laboratory experiments. Blueberries (*Vaccinium* spp.) are included to serve as a reference point for the pest performance on a preferred and suitable commercial crop. Boxplots provide the data for seven biological replicates per fruit species, each containing six to 14 berries depending on host species size: (**A**) Number of eggs laid on intact field-collected early spring host fruits; (**B**) Egg-to-adult survival; (**C**) Developmental time of *D. suzukii* adults emerged from the same fruits. Letters indicate statistical differences (*p* < 0.05) after Tukey’s multiple comparison test.

In no-choice laboratory trials, there was a significant difference among the host species for the number of *D. suzukii* eggs that were found in the fruits (Figure 4A) (GLM, negative binomial distribution: *χ*² _(5)_ = 81.25, *p* = 4 × 10^−16^). Compared with other early alternative hosts, *A. japonica* was infested by a significantly higher number of eggs per fruit. *Skimmia japonica* and *H. helix* had the lowest number of eggs per fruit (Figure 4A). The number of *D. suzukii* adults emerging from fruits was also significantly different between fruit species (GLM, quasibinomial distribution: *F*_(5, 36)_ = 15.27, *p* = 4 × 10^−8^). In *Vaccinium* spp. and *E. x ebbingei* species, about 30% of the eggs developed into adults, whereas the other species had a very low egg-to-adult survival (between 0 and 12%), including *A. japonica* with 5.7% emergence of *D. suzukii* adults (Figure 4B). The developmental time was significantly influenced by the fruit species (ANCOVA, normal distribution: *F*_(4, 21)_ = 26.80, *p* = 5 × 10^−8^): *D. suzukii* developing on *Vaccinium* spp. and *E. x ebbingei* had the shortest developmental time whereas those developing on *A. japonica* took longest (Figure 4C).

**Figure 5:**
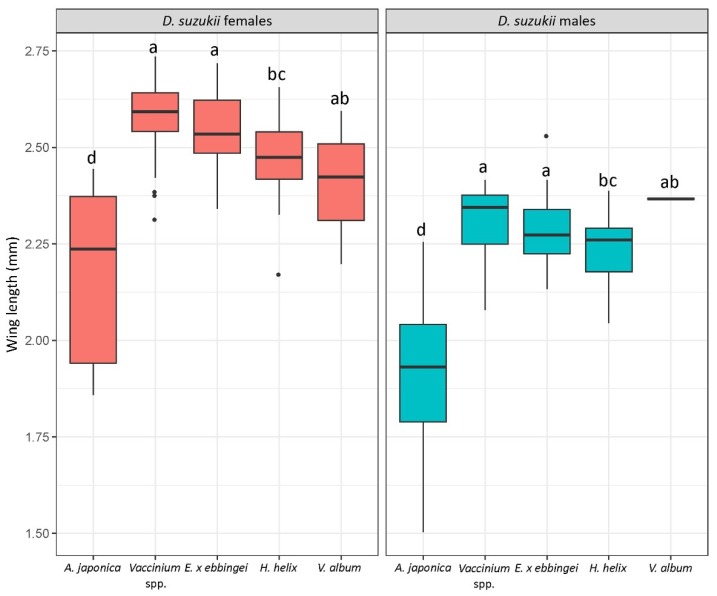
Wing length of *D. suzukii* adults emerged from the same above-mentioned fruits. Letters indicate statistical differences (*p* < 0.05) after Tukey’s multiple comparison test.

There was no significant interaction of fruit species and number of eggs on the developmental time (ANCOVA, normal distribution: *F*_(4, 21)_ = 1.38, *p* = 0.27). Wing length of the emerged *D. suzukii* adults significantly differed between fruits (ANOVA: *F*_(4,226)_ = 73.96, *p* < 2 × 10^−16^) and insect sex (ANOVA: *F*_(1,226)_ = 313.43, *p* < 2 × 10^−16^). Adults emerged from *A. japonica* fruits were the smallest by far, followed by adults emerged from *H. helix*. Males were significantly smaller than females (Figure 5). The interaction between host fruit species and sex was not significant (ANOVA: *F*_(4,226)_ = 1.38, *p* = 0.24).

### 3.4. Detection of D. suzukii Gravid Females and Collection of the First Infested Early Spring Host Fruits Are Concomitant

**Figure 6:**
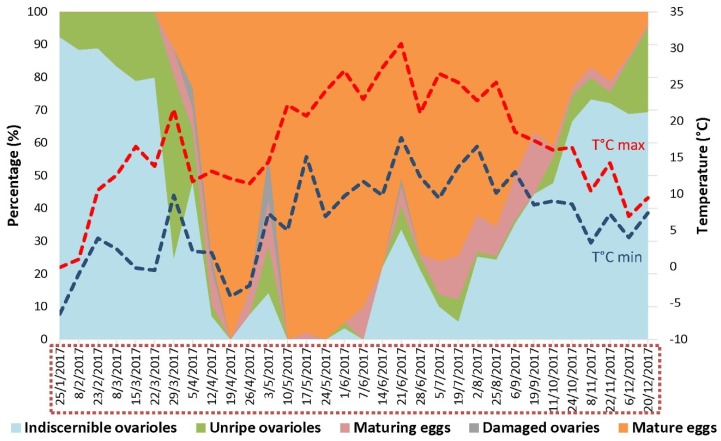
Seasonal reproductive status of dissected *D. suzukii* females captured in the field from January to December 2017. Reproductive biology is represented together with the minimum (blue dotted line) and maximum (red dotted line) daily temperatures in °C in the study area, The Netherlands. The red dotted rectangle around the dates refers to the period during which field-captured *D. suzukii* individuals were also assessed for their phenotype (see Figure 7).

**Figure 7:**
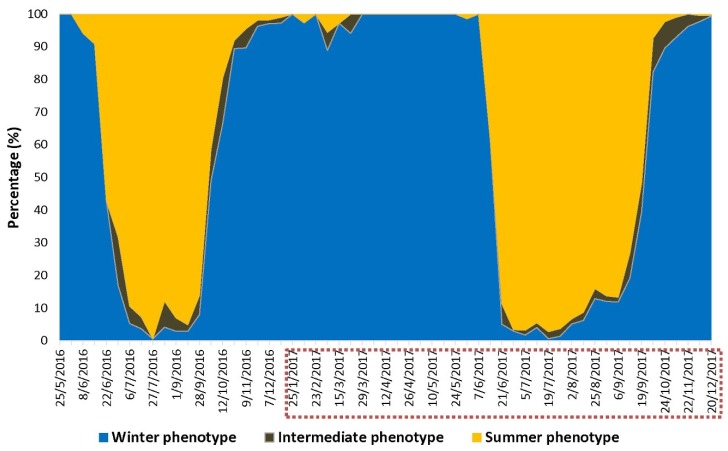
Ratio of phenotypes (summer, winter or intermediate morphs) of field-captured *D. suzukii* individuals during the sampling period May 2016–December 2017. The red dotted rectangle around some of the dates represents the period during which seasonal reproductive status of *D. suzukii* females captured in the field was assessed (see Figure 6).

The assessment of *D. suzukii* seasonal reproductive biology revealed a continuous period of reproductive activity from late March until late December (Figure 6). More than 50% of females captured between mid-April and September were gravid. From September onwards, a substantial fraction of females entered reproductive diapause. This coincided with the transition from trapping predominantly summer morphs to starting to capture substantial numbers of winter morphs (Figure 7).

While we continuously captured flies, even in very high numbers until the end of November, the percentage of reproductively active females gradually declined from 50% in mid-September to 3.5% by the end of December. From mid-October onwards, almost all captured flies were winter morphs. From January until March, no females bearing mature eggs were recorded. At all collection sites, the first mature females were detected at the end of March (87 DD) (Figure 6) and resumed oviposition as early as 1 April (104 DD) on early spring hosts, in particular *A. japonica*. Indeed, 4.2% of the sampled *A. japonica* fruits that had been collected at this date were infested by the pest (Figure 3). All 320 fruits of *A. japonica* fruits picked on 15 and 26 March were free from *D. suzukii* eggs.

### 3.5. Overwintered D. suzukii Females Are Likely the Main Contributors of the Infestation in the First Commercial Fruits

According to our temperature model, *D. suzukii* eggs that had been laid in any early host fruit from 1 April onwards were predicted to emerge as adults from 24 May (318 DD) onwards. When the early host plants would be the starting point of the seasonal buildup of flies, we expected the switch point from the overwintered winter morphs to the first generation of summer morphs to occur around the end of May–beginning of June.

The actual occurrence of the first *D. suzukii* summer-morph offspring was observed in mid-June for both 2016 and 2017 (Figure 7). The switch point from winter morph to summer morph individuals was recorded between the 15 and 22 June in 2016 (516–581 DD) and between the 14 and 21 June in 2017 (528–617 DD). According to the temperature model, these first summer morph flies had probably emerged from eggs laid between 26 May and 1 June in 2016 (307–371 DD) and between 24 May and 31 May in 2017 (318–401 DD). In the study area, the first susceptible commercial cherry crops were available to the pest from the end of May onwards (varieties: Earlise and Burlat). Indeed, the first *D. suzukii* eggs were identified on 1 June in 2016 (371 DD) and 27 May in 2017 (358 DD).

Potentially the first generation of *D. suzukii* summer-morphs could have emerged in time from early host plants to infest the first cherries. However, in practice, these (summer morph) flies were not detected by our monitoring program, as we only collected winter morphs during this period. Moreover, the flies that emerged from early host plants were characterized by a poor fitness and a rather low success of emergence. Taken together, these findings suggest that the first generation of *D. suzukii* summer-morph individuals mostly emerged from summer fruits, i.e., wild cherries, cultivated cherries and any other highly suitable wild hosts occurring at the same time as cherry crops. They originated from eggs that had been laid by overwintered winter-morph females.

## 4. Discussion

This study aims to better understand the seasonal biology and population dynamics of *D. suzukii* from early to late spring. It addresses important questions about the contribution of the early spring hosts to the seasonal buildup of the population and brings to light the crucial role of *D. suzukii* winter survivors in the infestation of the first cherry crops in the early season. Indeed, our results reveal that, as soon as the overwintered females break reproductive dormancy, they continuously lay eggs in a range of early spring host plants. Few summer-morph adults emerge from these unfavorable early spring hosts characterized by a low egg-to-adult survival. Furthermore, the few fruit flies that emerge tend to do so rather late in spring and are not detected by the monitoring programs, indicating that they are probably not the main contributors to the first infestation of commercial cherry fruits. Instead, our field monitoring data show that it is predominantly the overwintered *D. suzukii* females that infest the earliest ripening cherries, not their offspring from early spring hosts. These overwintered females live long enough to infest these cherry crops and any other suitable wild fruits occurring at the same time, leading to the development of the first generation of *D. suzukii* summer-morphs and the population peak recorded in late spring. This scenario is supported by all findings presented in this study.

The seasonal reproductive biology of *D. suzukii* in the Netherlands supports previous studies showing that *D. suzukii* females are reproductively active from early spring until late autumn, and the most stringent bottleneck for *D. suzukii* populations is from January to March in temperate climates [13,21,36,37,38]. In our study, females were found to resume oogenesis later than in Italy and North America where overwintered *D. suzukii* females start reproduction as early as 50 DD [13,38]. These variations might be explained by factors including differences in winter conditions over years, microclimate, various agricultural and geographical contexts, genetic variability and trapping techniques. Though correlated with physiological time, the reproductive potential of *D. suzukii* females in early spring is probably not only influenced by temperature. The contribution of other parameters such as humidity and resource availability needs further investigation [38,39,40].

Our field assays show that all plant species monitored in 2017 were used by *D. suzukii* for oviposition, confirming the wide range of potential host plants that the flies can infest [15,18,27,28,29]. Trapping of the first gravid females in late March closely corresponded with finding the first eggs on early spring hosts. Fruits of *A. japonica* had a high infestation rate compared with other studied species and were increasingly infested by the pest from early to late spring. This early spring host was reported several times as a suitable host in the field in Japan [22,29]. Based on the various collection sites and the great number of fruits that were sampled over time we consider our study representative for early spring hosts’ availability for *D. suzukii* oviposition in the Netherlands. Importantly, though, while several of the early host plants served as oviposition sites during spring, only few allowed for the complete development of the pest. In early spring, in 2017, only *A. japonica* and *E. x ebbingei* yielded some offspring. In 2016, a few more plant species were found to support the development of the pest from egg to adult but later in spring. This indicates some variations in the suitability of alternative hosts over the season and over years. However, for all plant species, the egg-to-adult survival was relatively low and variable.

The laboratory assays were in line with these observations. A few more host species were able to support the pest during its development from egg to the adult stage under controlled conditions, in accordance with previous research [13,15,18,27]. The few *D. suzukii* adults that emerged from the early hosts had a poor fitness as indicated by their longer developmental time and their smaller size compared with *D. suzukii* emerging from blueberries. *Elaeagnus x ebbingei* was an exception and, in laboratory trials, this natural host was quite similar to blueberries for all tested parameters. However, fruits of *E. x ebbingei* are not very common in the study area as this species is mostly used as a hedge plant and is frequently pruned. Although all collected fruit species used for the laboratory trials were kept in containers at a high humidity, it should be acknowledged that some of them might have been underestimated for their capacity to host the pest from egg to adult stage due to their sensitivity to desiccation and the fact that they can become less suitable to the pest after picking. This is especially the case for *H. helix* [41].

Interestingly, field observations over time suggest that early host plants become more suitable to the pest later during spring. Infested *A. japonica* fruits collected on 17 April 2017 were the only early spring hosts from which flies emerged, but at a later collection date, on 8 May, the pest also completed its life cycle on an additional species, *E. x ebbingei*. In 2016, similar patterns were observed for *V. album*. This wild host was collected regularly from March until June and the first emergence of *D. suzukii* adults was recorded from fruits that had been collected on 12 May, while field-monitoring data showed that *D. suzukii* was present and trapped on-site from March onwards [42]. Thus, the pest and host presence were not limiting factors in the infestation process. These observations suggest that in early spring, alternative hosts may be used for the pest oviposition but are unsuitable for the pest development before mid to late spring. This phenomenon could be explained by physiological changes that characterize fruit development such as sugar content increase and/or penetration force decrease with fruit ripening, favoring the pest oviposition and larval development [43,44,45]. Thus, the higher *D. suzukii* adults’ emergence from non-crop hosts observed later in spring could result from an improved quality of early host plants. It could also relate to a higher viability of the eggs/zygote due to potential remating of overwintered females.

Overall, the contribution of the early spring host plants to the seasonal buildup of *D. suzukii* seemed to be relatively minor since the first generation of summer-morphs came mostly from eggs laid by overwintered (winter-morph) females on summer fruits, i.e., any cultivated and wild fruits available at the same time as cherry crops. The absence of summer-morphs emerged from early spring hosts in the monitoring program could be due to a lack of monitoring traps located in private gardens, parks etc. characterized by the presence of natural hosts. It could also relate to the fact that bait attraction depends on the physiological state of *D. suzukii* and on the attractant used in the traps [46]. In that respect, the fermentation-based traps used in this study might have been little attractive to newly-emerged summer-morph adults. Alternatively, their absence of the monitoring program may be due to the small number of these flies, compared with the winter survivors, and their poor fitness. Our findings are in support of the latter since the traps used in our monitoring program were located in orchards in close vicinity to urban areas and forested environments. Considering the high dispersal rates of *D. suzukii* over long distances, it seems improbable that the traps’ location has been a limiting factor [47,48,49,50]. Moreover, the traps attracted *D. suzukii* throughout the year, and both summer and winter morphs. Thus, the late emergence of summer morphs in the season reinforces the proposed scenario that the main source for infestation of the early commercial crops is in fact the winter survivors.

Overwintered *D. suzukii* females are thought to store sperm from autumn matings to counteract the winter bottleneck that may result in a scarcity of mature males in early spring. This strategy would allow them to resume oviposition when they exit reproductive diapause in early spring, without needing to mate again [13,19,28,51]. In Italy, during the winter bottleneck at least 30% of the females contained sperm, indicating that they would be able to produce fertilized eggs right after the reproductive diapause [13]. Our study confirms that overwintered *D. suzukii* females are able to live very long [21,52] and supports earlier research on *Drosophila*, showing that flies have a much longer lifespan when raised at low temperature [53]. Together with previous observations, our findings call for further investigation to get a better insight into how long the overwintered females can survive after a cold treatment and over which period they can produce viable offspring, relying on matings performed before the cold exposure.

Previous studies have advised to target the pest in early spring, for example in applying bait sprays and releasing sterile males and/or biological control agents, to take advantage of low population levels and low reproductive potential during this bottleneck period [13,19,54]. Our results highlight other aspects that should be taken into account to efficiently target the pest. Indeed, they imply that the Sterile Insect Technique (SIT) in spring might not be as promising as we would like. They also show that the key starting point for further seasonal build up are overwintered *D. suzukii* females probably coming from sites in which *D. suzukii* reproduced before winter. Considering that a major part of the late reproduction season occurs in habitats that cannot be sprayed with insecticides, sanitation on a large scale may not be efficient to suppress the future winter survivors [15]. Instead, classical biological control through the release of natural enemies might provide a more reliable solution for an area-wide control approach [55,56,57].

## 5. Conclusions

In conclusion, the role of early spring oviposition host plants has probably been overestimated with respect to the infestation of the first fruit crops of the season. However, they may still contribute to a certain extent to the population increase, especially from mid-spring onwards. One early spring host species, *A. japonica*, was characterized by very high oviposition rates and low egg-to-adult survival. The variable success of adult emergence observed in preliminary assays suggests that *A. japonica* does not serve as a “dead-end” host plant that stimulates the pest oviposition but does not allow full larval development. However, this plant species might be useful for monitoring purposes. Trap or monitoring plants could be valuable in the frame of an IPM plan since they could be planted around orchards; especially when these plants are more attractive than the (early stages of) commercial crops, this could limit the number of pesticides sprays [27]. Our analysis provides useful information about the seasonal biology of *D. suzukii* that can help develop an integrative management strategy for this new invasive pest species. The data presented could be implemented into population models in order to help better forecast the pest population dynamics in spring and to design effective and efficient IPM techniques. There is probably no stand-alone control solution for this new pest species and it is, thus, crucial to further study the interaction and complementarity of existing management tools.

## Figures and Tables

**Table 1:** Fruit seasonality of early spring hosts of *D. suzukii* and commercial crops in the study area during 2016 and 2017

Plant Species	Jan	Feb	Mar	Apr	May	Jun	Jul	Aug	Sep	Oct	Nov	Dec
*Aucuba japonica*												
*Elaeagnus x ebbingei*												
*Skimmia japonica*												
*Hedera helix*												
*Viscum album*												
Cherry crops												
Soft fruit crops												
Grape crops												

**Table 2:** Number (No.) of *D. suzukii* adults emerged from early host plants collected between March and June 2016 in the study area

Plant Species	No. Sampled Locations	No. Collected Fruits	No. *D. suzukii* Adults Reared	Infestation Rate (%)
*Cotoneaster* spp.	2	470	0	0.0
*Elaeagnus x ebbingei*	1	758	38	5.0
*Hedera helix*	4	550	0	0.0
*Aucuba japonica*	7	791	61	7.7
*Skimmia japonica*	1	860	6	0.7
*Viscum album*	1	1870	55	2.9

**Table 3:** Natural infestation of field-collected early spring fruits and successful *D. suzukii* adult emergence under laboratory conditions. Results are presented as the percentage of collected fruits naturally infested by *D. suzukii* eggs at two time points in spring 2017. The egg-to-adult survival is assessed by calculating the ratio between the total number of eggs laid on the incubated fruits and the number of flies emerged from these fruits.

		No. Collected Fruits	% Infested Fruits	Incubation in the Laboratory			
				No. Infested Fruits ^1^	No. Eggs	No. Emerged Flies	Hatch Rate
**17 April**	*Aucuba japonica*	282	35.1	48	87	3	3.4
	*Elaeagnus x ebbingei*	213	2.3	5	5	0	0.0
	*Skimmia japonica*	308	1.0	3	6	0	0.0
	*Hedera helix*	1472	0.2	3	3	0	0.0
	*Viscum album*	374	0.0	0	0	0	0.0
**8 May**	*Aucuba japonica*	321	53.0	97	189	20	10.6
	*Elaeagnus x ebbingei*	322	10.9	35	43	16	37.2
	*Skimmia japonica*	347	0.9	3	3	0	0.0
	*Hedera helix*	1400	0.2	3	3	0	0.0
	*Viscum album*	412	0.0	0	0	0	0.0

^1^ For each host species, all infested fruits were incubated in the laboratory except for *Aucuba japonica* where approximately half of the infested fruits was incubated under field conditions.

**Table 4:** Successful *D. suzukii* adult emergence on naturally infested *A. japonica* fruits incubated either in the field or under laboratory conditions during 2017.

	Field Incubation				Laboratory Incubation			
Incubation Date ^1^	No. Infested Fruits	No. Eggs	No. Emerged Adults	Hatch Rate	No. Infested Fruits	NO. EGGS	No. Emerged Adults	Hatch Rate
10 April	25	40	0	0.0	2	40	1	2.5
17 April	49	94	0	0.0	48	87	3	3.4
23 April	63	132	2	1.5	62	128	4	3.1
1 May	73	158	5	3.2	70	149	4	2.7
8 May	73	101	5	5	97	189	20	10.6
15 May	135	340	42	12.4	127	323	84	26
22 May	121	441	10	2.3	116	414	15	3.6
29 May	79	73	0	0	179	81	0	0
**Total**	**618**	**1379**	**64**	**4.6**	**724**	**1411**	**131**	**9.3**

^1^ All naturally infested *A. japonica* fruits collected between 10 April and 29 May were divided in approximately two halves and incubated either in the field or in the laboratory. The percentage of collected fruits naturally infested by *D. suzukii* eggs at each collection date is presented in Figure 3.

## Supplementary Materials

The following are available online at http://www.mdpi.com/2075-4450/9/4/145/s1, Table S1: Characteristics of the non-crop host species tested in the study in 2016 and 2017, Table S2: Collection date, location, habitat and number of fruits sampled for each non-crop host species in 2017, The Netherlands, Table S3: Additional locations and habitats of weekly sampled *Aucuba japonica* fruits in the study area, The Netherlands, during 2017, Table S4: Locations and monitoring sites of *D. suzukii* adults in the study area from 2016 to 2017, The Netherlands, Table S5: Number (No.) of *D. suzukii* adults emerged from *Viscum album* fruits collected in the study area, Hedel, The Netherlands, during 2016.
