# Supplementary material for: Overwintered Drosophila suzukii Are the Main Source for Infestations of the First Fruit Crops of the Season
Source: Insects. 2018 Oct 22;9(4):145. doi: 10.3390/insects9040145 (PMC6315960; doi:10.3390/insects9040145)
Supplement: Supplementary file 1 [file insects-09-00145-s001.pdf]

Overwintered *Drosophila suzukii* Are the Main Source for Infestations of the First Fruit Crops of the Season

Aurore D. C. Panel [1,2], Laura Zeeman [3,4], Bart J. van der Sluis [4], Peter van Elk [4], Bart A. Pannebakker [2], Bregje Wertheim [1] and Herman H. M. Helsen [4,*]

[1] Groningen Institute for Evolutionary Life Sciences, University of Groningen, Nijenborgh 7, 9700 CC Groningen, The Netherlands; a.d.c.panel@rug.nl (A.D.C.P.); b.wertheim@rug.nl (B.W.)
[2] Wageningen University & Research, Laboratory of Genetics, PO Box 16, 6700 AA Wageningen, The Netherlands; bart.pannebakker@wur.nl
[3] Wageningen University & Research, Laboratory of Entomology, PO Box 16, 6700 AA Wageningen, The Netherlands; laura.zeeman@wur.nl
[4] Wageningen University & Research, Field Crops, 6670 AE Zetten, The Netherlands; bart.vandersluis@wur.nl (B.J.v.d.S.); peter.vanelk@wur.nl (P.v.E.)
* Correspondence: herman.helsen@wur.nl; Tel.: +31-488-473-754

Electronic Supplementary Material

Table S1. Characteristics of the non-crop host species tested in the study in 2016 and 2017.

Plant species	Family	Geographical origin	Used as ornamental plant	Native in temperate Europe	Management of sampled plant species
Aucuba japonica	Garryaceae	Asia	X		Planted
Elaeagnus x ebbingei	Elaeagnaceae	Europe, North America	X	X	Planted
Skimmia japonica	Rutaceae	Asia	X		Planted
Hedera helix	Araliaceae	Europe and Western Asia	X	X	Planted, spontaneous
Viscum album	Santalaceae	Europe, Western and Southern Asia		X	Planted
Cotoneaster spp.	Rosaceae	Central Europe	X	X	Planted

Table S2. Collection date, location, habitat and number of fruits sampled for each non-crop host species in 2017, The Netherlands.

Collection date	Plant species	Location	Site[a]	Latitude	Longitude	Habitat	No fruits
April, 17	*Aucuba japonica*	Wageningen	Plantsoen	51.97907957	5.65587519	Urban area, private gardens	65
		Wageningen	Arboretum	51.96741361	5.67643287	Woody plants, bushes	50
		Wageningen	Arboretum	51.96549334	5.69108201	Woody plants, bushes	14
		Wageningen	Roghorst	51.979784	5.668604	Urban area, private gardens	40
		Zederik	Smalzijde21A	51.8925	5.0137	Private gardens, grassland	53
		Gouda	KleinAmerica	52.012905	4.715813	Urban area, private gardens	60
	Elaeagnus x ebbingei	Gouda	Baden Powellplantsoen	52.008203	4.737165	Urban area, private gardens	213
	Skimmia japonica	Wageningen	Nassauweg	51.966952	5.674142	Urban area, private gardens	81
		Wageningen	Hoogvliet	51.965185	5.658808	Urban area, private gardens	65
		Wageningen	Arboretum	51.967582	5.677586	Woody plants, bushes	109
		Zederik	Smalzijde21A	51.8925	5.0137	Private gardens, grassland	53
	Hedera helix	Wageningen	Boterstraat	51.96409104	5.66415024	Urban area, private gardens	119
		Wageningen	Wallepad	51.96417489	5.66569162	Urban area,	633

						private gardens	
		Wageningen	Mennonietenweg	51.965864	5.656395	Urban area, private gardens	320
		Gouda	Jan de Boerplein	52.009644	4.738019	Urban area, private gardens	400
	Viscum album	Hedel	Hedel	51.748802	5.241822	Mistletoe plants on apple trees, organic cherry orchard	374
May, 8	*Aucuba japonica*	Wageningen	Roghorst*	51.978688	5.665533	Urban area, private gardens	80
		Wageningen	Arboretum	51.96741361	5.67643287	Woody plants, bushes	60
		Wageningen	Plantsoen	51.97907957	5.65587519	Urban area, private gardens	63
		Gouda	KleinAmerica	52.012905	4.715813	Urban area, private gardens	53
		Zederik	Smalzijde21A*	51.8925	5.0137	Private gardens, grassland	65
	Elaeagnus x ebbingei	Ede	Jan Tooroplaan	52.02570898	5.66827097	Urban area, private gardens	22
		Gouda	Baden Powellplantsoen*	52.008203	4.737165	Urban area, private gardens	300
	Skimmia japonica	Zederik	Smalzijde21A	51.8925	5.0137	Private gardens, grassland	60
		Wageningen	Nassauweg	51.966952	5.674142	Urban area, private gardens	71

		Wageningen	Hoogvliet	51.965185	5.658808	Urban area, private gardens	154
		Wageningen	Arboretum*	51.967582	5.677586	Woody plants, bushes	62
	Hedera helix	Ede	Frans Halslaan	52.01697278	5.65557069	Urban area, private gardens	180
		Wageningen	Wallepad*	51.96417489	5.66569162	Urban area, private gardens	500
		Gouda	Jan de Boerplein	52.009644	4.738019	Urban area, private gardens	720
	Viscum album	Hedel	Hedel*	51.748802	5.241822	Mistletoe plants on apple trees, organic cherry orchard	412

[a] Single asterisk indicates the sites from which non-crop fruits used for the no-choice laboratory trials were sampled.

Table S3. Additional locations and habitats of weekly sampled *Aucuba japonica* fruits in the study area, The Netherlands, during 2017.

Area	Location	Site	Latitude	Longitude	Habitat
Zederik	Zederik	Smalzijde21A	51.8925	5.0137	Private gardens, grassland
Gouda	Gouda	KleinAmerica	52.012905	4.715813	Urban area, private gardens
Wageningen	Bennekom	Vossenweg	52.008014	5.674786	Urban area, private gardens
	Bennekom	Selterskampweg	51.999225	5.685515	
	Bennekom	Diedenweg	51.996014	5.684019	
	Wageningen	Arboretum 1	51.96549334	5.69108201	Woody plants, bushes
	Wageningen	Arboretum 2	51.96741361	5.67643287	
	Wageningen	Belmonte	51.96847818	5.69365962	Urban area, private gardens
	Wageningen	Bevrijdingskerk	51.96953857	5.67260378	
	Wageningen	Roghorst 1	51.979819	5.668446	
	Wageningen	Roghorst 2	51.979784	5.668604	
	Wageningen	Roghorst 3	51.979715	5.664474	
	Wageningen	Roghorst 4	51.9786880	5.665533	
	Wageningen	Hinkeloordseweg	51.96646397	5.67499847	
	Wageningen	Nassauweg	51.96691077	5.67437355	
	Wageningen	Plantsoen	51.97907957	5.65587519	
	Wageningen	Tarthorst	51.979659	5.659883	

Table S4. Locations and monitoring sites of *D. suzukii* adults in the study area from 2016 to 2017, The Netherlands.

Site location	Trap code	Collection site	Latitude	Longitude	Habitat typology
Kesteren	1	Cherry orchard	51.92987456	5.55133357	Cherry orchard
	2	Cherry orchard	51.9327303	5.54525957	
	3	Cherry orchard	51.93386368	5.54411641	
	4	Hedgerow	51.93401558	5.5539907	
	5	Private garden	51.93423394	5.55469314	
Wageningen	6	Apple tree	51.97442993	5.69791237	Vineyard and wood
	7	Bushes	51.97429792	5.69559795	
Randwijk	8	Berry orchard	51.93749487	5.7084881	Cherry and berry orchard
	9	Cherry orchard	51.93748266	5.70439969	

Table S5. Number (No.) of *D. suzukii* adults emerged from *Viscum album* fruits collected in the study area, Hedel, The Netherlands, during 2016.

**Collection date**	**No. of collected fruits**	**No. of *D.suzukii* adults reared**	**Infestation rate (%)**
March, 30	180	0	0
April, 28	140	0	0
May, 4	200	0	0
May, 12	727	7	1
June, 2	402	14	3.5
June, 9	221	34	15.4
